# Balance between solitude and socializing: everyday solitude time both benefits and harms well-being

**DOI:** 10.1038/s41598-023-44507-7

**Published:** 2023-12-05

**Authors:** Netta Weinstein, Matti Vuorre, Mark Adams, Thuy-vy Nguyen

**Affiliations:** 1 University of Reading, Reading, UK; 2 Tilburg University, Tilburg, The Netherlands; 3 Durham University, Durham, UK

**Keywords:** Human behaviour, Psychology

## Abstract

**Abstract:**

Two literatures argue that time alone is harmful (i.e., isolation) and valuable (i.e., positive solitude). We explored whether people benefit from a balance between their daily solitude and social time, such that having ‘right’ quantities of both maximizes well-being. Participants (*n* = 178) completed a 21-day diary study, which quantified solitude time in hours through reconstructing daily events. This procedure minimized retrospective bias and tested natural variations across time. There was no evidence for a one-size-fits-all ‘optimal balance’ between solitude and social time. Linear effects suggested that people were lonelier and less satisfied on days in which they spent more hours in solitude. These detrimental relations were nullified or reduced when daily solitude was autonomous (choiceful) and did not accumulate across days; those who were generally alone more were not, on the whole, lonelier. On days in which people spent more time alone they felt less stress and greater autonomy satisfaction (volitional, authentic, and free from pressure). These benefits were cumulative; those who spent more time alone across the span of the study were less stressed and more autonomy satisfied overall. Solitude time risks lowering well-being on some metrics but may hold key advantages to other aspects of well-being.

**Protocol registration:**

The stage 1 protocol for this Registered Report was accepted in principle on June 1, 2022. The protocol, as accepted by the journal, can be found at: 10.17605/OSF.IO/5KXQ3.

## Introduction

The COVID-19 outbreak made eminently salient the role that solitude plays in daily life. The effects of increased solitude in our lives were richly varied for reasons still unknown, with widespread reports of loneliness^1,2^ alongside those describing well-being^3^, relaxation^4^, creativity^5^, and productivity^6^. Considering this recent work in its sum, we might observe individuals responding in a host of ways to what was, in effect, a lasting disruption to their otherwise normative patterns of daily solitude and socializing. It speaks, indirectly, to certain unanswered questions: how do people respond to time spent in solitude, are there benefits to greater amounts of daily solitude, and is there such a thing as too much solitude? These questions reflect the goal of a nascent body of work: that of understanding *balance* between time spent alone and time spent socializing with others. The proposed study tested balance operationalized as daily hours alone and interacting with others to examine both within- and between- person reactions to the proportion of daily time spent in solitude.

The question of balance is at the heart of the study of time spent alone and speaks to what has been termed the *paradox of solitude*^7,8^, that being alone has seemingly contradictory well-being effects across literatures. On one hand, studies of positive solitude demonstrate affordances of this time that yield a number of well-being outcomes^9^. On the other hand, studies on isolation conclude that time spent alone is antithetical to the social nature of humans and detrimental to well-being^10^ (see also review^11^). A recent study of aloneliness—understood as the desire to have more time alone—integrates these two approaches to argue that just as loneliness arises from insufficient social context, aloneliness is experienced when individuals do not have enough time in solitude^7^. In short, we may need both—time alone *and* time with others—in order to experience the full range of well-being benefits.

One set of observations related to solitude is that time spent alone can yield well-being benefits under certain conditions. Even within the earliest views of solitude, Western and Eastern spiritual leaders espoused the importance of sufficient periods of alone time for spiritual growth^12,13^. Hermetic traditions have also prized solitude as key to a life well-lived, holding the view that by spending time alone individuals can gain self-knowledge and self-connection that is difficult or impossible in the company of others^14^. Research studies of daily solitude have explored positive affordances of solitude^9,15^, with mounting evidence for low-arousal positive affect such as feeling less stressed, and more peaceful and relaxed (also termed, deactivation effect^16,17^). Along with this affective regulatory benefit, solitude may provide other affordances that satisfy needs^9,18,19^, including fostering self-connection, or feeling autonomy need-satisfied through volitional and self-congruent action and experience^20^.

Though time spent alone can offer well-being benefits, there is substantial evidence that for many or most, large amounts of solitude can undermine well-being. Work in this area has focused on unusual circumstances of solitude such as solitary confinement^21,22^, and polar as well as space exploration^23^. However, everyday experiences of time spent alone under more typical circumstances can also have broad-reaching well-being costs^24^. Perhaps the most prominent outcome of too much time spent alone is loneliness, the perceived discrepancy between desired and actual social connection (i.e., that there is less social connection than desired)^25,26^, which itself has significant and lasting mental and physical health costs^27,28^.

Taken together, it appears that some solitude may conduce well-being, but that a tipping point may exist such that quantities of solitude beyond an ideal, moderate point may be detrimental. Whereas isolation—excessive solitude—is aversive^29^, insufficient amounts of solitude may also be detrimental, and lead individuals to seek out more time alone^7^. This view of balance can be observed in people’s behaviors. For example, long-distance hikers and naturalists report that solitude is a beneficial quality of the activity^30,31^, but their time alone is intentionally balanced by intermittent social interactions during treks^32^.

Despite indirect evidence that time plays a key role, it may not be possible to estimate balance simply through an assessment of time spent alone and with others. Alongside the amount of solitude, the motivation for it—those reasons that lead individuals to be spending time alone—may fundamentally shape its shifting impact on well-being^33,34^. To characterize motivation and understand its influence on those spending time alone, researchers have identified that two forms of autonomous (i.e., self-driven) motivation play an important role: self-determined motivation (pursuit of solitude because one values and enjoys the experiences)^35^, and choiceful motivation (pursuit of solitude that is freely chosen^17^).

The two constructs conceptually overlap and reflect the same underlying quality of autonomous motivation^20^: pursuing solitude for self-driven and volitional reasons, rather than because one has no choice or other options are unappealing or aversive^20^. Autonomous motivation for solitude is therefore seen as definitional of positive solitude in literatures that place less emphasis on time spent alone^33^, and its absence has been definitional to isolation. For example, the term active-isolation has been used to refer to solitude that, while not necessarily extensive, is caused by external circumstances such as being ostracized or excluded^34^. In addition, the term social withdrawal has been used to refer to solitude driven by an anxious avoidance of peers, a distinctly non-autonomous motivation^36^.

These autonomous forms of motivation for solitude have been related to well-being generally^37,38^, and in daily diary studies showing that they correlate with everyday well-being^17,39,40^. In cross-sectional research, amount of time spent in solitude has also been linked to reduced life satisfaction linearly (non-linear effects were not tested), but only for those who have less self-determined motivation for solitude^41^. As well, leading solitude theorists have reasoned that as people desire more solitude, they benefit more from their time alone. Integrating this view with the motivation for solitude literature, it is likely that autonomous (self-determined and choiceful) motivation for solitude drives more positive solitude time, and may mitigate costs of large amounts of solitude.

In short, the question of balance in solitude may be a simple one: What proportion of our time should be spent alone? Yet nuance arises when testing this issue in depth. For example, balance may exist at the day level or as a function of time spent alone across a larger span of time. It may also produce non-linear changes in one or multiple well-being indicators. Furthermore, the point of balance may vary, and may be best understood in relation to a meaningful moderator, such as autonomous motivation for solitude^35^. Our general aim was to explore the question robustly by considering these issues. To do so, we operationalized balance in terms of the tipping point at which the linear well-being benefits of having no-to-some solitude shift into a different pattern of linear well-being costs of having some-to-much solitude.

We focused on adults aged 35 years and over, those participants who had completed transition from young adulthood to adulthood^42,43^. During this age range individuals begin to orient to building careers, families, and adult friendships, and have finished negotiating independence from family-of-origin relationships^44^. In Erikson’s psychodynamic model, individuals are undertaking or have completed the challenge of building intimate relationships or experiencing feelings of isolation^45^. Previous research focusing on this age range found correlations between individual differences in self-determined motivation and overall well-being, which were stable across time of a multi-wave study but not cumulative. Here, we extend the research to examine both time and motivation simultaneously as they influence well-being on a daily level. We set out to test basic Research Questions (RQ) and their corresponding hypotheses (H):

(RQ 1) Is there an average inflection or tipping point (i.e., maxima or minima) wherein the relationship between the proportional time spent in solitude and well-being outcomes shifts?

(H1) We hypothesized that quadratic slopes would be present for proportional time spent in solitude such that:

(H1a) When below the local maxima/minima, higher proportional time in solitude would relate to less aloneliness, less perceived stress, higher autonomy and day satisfaction.

(H1b) When above the local maxima/minima, higher proportional time in solitude would relate to more loneliness and perceived stress, lower autonomy and day satisfaction.

(RQ 2) Can balance be conceptualized in terms of average time in solitude and social interactions across a longer period (specifically, 21 days), and/or as a phenomenon that occurs at the daily level such that each day has its own balance of solitude and social interaction time? There is no literature to strongly suggest that daily or stable effects would dominate, so we did not have a directional hypothesis related to this question; rather, we explored it through estimating quadratic effects of solitude time at both Level 1 (daily level) and Level 2 (individual level).

(RQ 3) Do the detrimental effects of high amounts of solitude differ as a function of autonomous motivations for solitude?

We hypothesized that:

(H2) Autonomous motivation for solitude (in the form of self-determined motivation at Level 2, and choiceful motivation at Level 1) would relate to higher daily well-being.

(H3) Days in which individuals endorse more choiceful motivation for solitude would show lower reduction in well-being when individuals spend more time in solitude above the local maxima/minima.

(H4) Individuals higher in self-determined motivation for solitude would show lower reduction in well-being when they spend more time in solitude daily above the local maxima/minima.

## Method

### Ethical approval

All methods were performed in accordance with relevant guidelines and regulations for conducting human subjects research. The proposed research complied with APA and BPS ethical regulations. Ethical approval for this study was attained by the Psychology Department’s Ethics Committee at the University of Reading before undertaking study procedures. Written informed consent and verbal assent was attained from all participants of the study before starting, and they were debriefed on study goals and reminded of data management processes at the end of the study. Participants could withdraw from the study at any point.

### Overall approach

This study employed methodological and analytic approaches from previous research on solitude, but not related to balance^7,46^, and from time-use balance studies in another domain of behavior (i.e., screentime^47^). Following on typical practices for daily diary designs^48,49^, brief measures were used where possible, to reduce participant burden.

### Participants and recruitment

We planned for participants to be 150 adults, in total, living in either the United Kingdom or the United States. The final sample of participants included 178 adults (79 men, 1 non-binary or genderqueer, 95 women, three did not answer; age mean: 47, IQR: 39–53) who completed 16.7 study days (IQR: 16–20) on average. Data collection took place between Sep. 1st, 2022 and Nov. 4th, 2022. We oversampled due to a miscount when recording the number of participants who had taken part in the study at that point. Three individuals did not complete the first survey, and we therefore could only include 175 individuals in analyses using Level-2 variables.

We sought participants aged 35 years or older to focus in on adult experiences with solitude and interpersonal relationships, and based on previous research on motivation in solitude in adulthood^50^. Participants were compensated financially up to £60 or $72 each for taking part in the study, in full, or partially as a function of days completed. This is higher than the planned amount of £36 or $40 because when we piloted the survey, we realized it took quite a bit longer than we had anticipated. Compensation was adjusted accordingly to align to living wage.

Power simulations were conducted using the ‘simr’ package (version 1.0.5) in R program to estimate power achieved to detect predicted main effects of 0.30 and predicted interaction effects of 0.20. A sample size of 150 participants with 21 observations per participant allowed us more than 80% power to detect significant fixed effects at 0.05 alpha levels. Approximately 150 participants were needed for any additional multilevel analysis to achieve an acceptable power of 0.80–0.90 and given up to 21 observations and a moderate effect size, but we also anticipated 20–40% data loss in these types of studies^51^. Our plan to collect up to 3150 data points was also based on recommendations to include a minimum of 1600–1700 data points for mixed effects models with an unclear prior effect size^52–54^.

We were successful in collecting 2967 data points. To recruit participants, we relied on Prolific after difficulties engaging participants for this intensive study using local in-person posters and social media adverts.

### Exclusion criteria

In line with our plans, participants were excluded from analyses if they completed fewer than three days of the daily diary study. With three or more days of daily diary responses, we anticipated minimum daily variability to test day-level effects alongside between-person predictors. The multilevel models planned are well-suited to handle missing data even for our participants who responded to a small portion of the study days^55^. We anticipated no other need to exclude participants.

### Procedures

All participants received a video chat initiation session designed to increase adherence across three weeks and vet individuals’ age and eligibility (namely, adult English speakers living in the US or UK). During this session, participants completed a brief set of questionnaires assessing demographic variables for reporting purposes, report on self-determined motivation for solitude, and receive verbal training on how to use the experience sampling method survey technology. Following this, a 21-day diary study using a fixed-interval design with surveys was completed in the evenings between 20:00 and 24:00. This survey examined daily time in solitude and socializing, and well-being outcomes. The approach is also used in other solitude work, and other investigations of daily well-being as a function of motivation^56^. It allowed us to (1) capture moments that naturally occur in the lives of individuals, (2) minimize retrospective bias, because responses can be measured in close temporal proximity to the occurrence of the events, (3) statistically model within- and between-person effects.

### Independent variables

#### Reported time spent alone and with others

Building on previous work (Goldilocks Effect^47^) that had operationalized balance in screen time by quantifying tipping points, we measured the number of hours spent alone and with other people each day. Because even daily diary measurements are subject to some retrospective bias^57^, this was done using a day reconstruction method^58^. Participants described each episode of their day, describing (1) each activity in which they engaged, (2) the time the activity began and ended, and (3) whether or not they were in solitude. To measure solitude, we applied a method used by Lay et al. (2018), which the authors based on a conceptualization of solitude in terms of the subjective experience of being alone without social interaction, regardless of whether others are around. Following a recent argument by Campbell and Ross^59^ that solitude does not take place when individuals are communicating digitally, and recent qualitative findings that adults see technology-mediated communication as antithetical to solitude^60^, we also measured both digitally mediated as well as in-person communications.

For each activity, we asked participants to report: (a) “Are you alone?” From this prompt they selected between ‘*yes *(*no one else is around*)’ or ‘*no *(*somebody/others are around*)’. We also measured interpersonal exchanges: (b) “Are you interacting with someone in-person (e.g., face-to-face conversation)?”, and (c) “Are you interacting with someone through technology (e.g., text or phone)?”. These two questions were paired with ‘*yes*’ or ‘*no*’ options. When participants reported they are alone and nobody else is around, they were shown (c: technology-mediated interaction) but not (b: in-person interaction). If responding ‘*no (somebody/others are around)*’, they were asked both (b) and (c). Building on Campbell and Ross (2021), and Lay et al. (2018), participants were coded as being in solitude (i.e., the absence of social interaction) only if they selected ‘*no*’ to both in-person and digital forms of communication. They were coded as being in non-solitude (i.e., social) when they say ‘*yes*’ to either form of communication. The total number of hours spent each day in solitude were calculated from the raw event time-span reports provided by participants, coded to the minute.

Daily balance was operationalized as the proportion of time spent alone relative to socializing.

Linear proportion of time spent in solitude (Ltime):

$$Linear \, proprotion \, of \, time \left(Ltime\right)= \frac{Hours \, solitude}{Hours \, solitude+Hours \, socializing}$$

Quadratic proportion of time spent in solitude (Qtime):

$$Quadratic \, proprotion \, of \, time \, \left(Qtime\right)=\left(Ltime\right)^2.$$

### Outcome measures

#### Treatment of all outcome measures

All outcome measures were paired with a scale ranging from 0 (*not at all*) to 6 (*extremely*) unless noted otherwise for consistency across the survey, and because 7-point Likert-type scales provide higher internal consistency and test–retest reliability than do measures with fewer scale points^1^.

#### Loneliness

We selected a measure of loneliness that was face valid and not confounded with either the quantity of social interaction or feelings of rejection (e.g., UCLA Loneliness Scale includes items such as “no one really knows me well”, “I am unhappy being so withdrawn”, and the three-item loneliness scale includes “how often do you feel you lack companionship?”^61,62^). Instead, participants completed a single item as a direct measure: “I felt lonely today”, as a common and face-valid way to assess the construct^63^. This approach is appealing to participants^64^, and allows them to consider what the term ‘loneliness’ means to them^46^.

#### Aloneliness

The newly developed and validated measure of aloneliness assesses a desire to be alone that stems from perceived discrepancy between actual and desired alone time, and is designed as a counterpoint to loneliness^7^. The scale has a one-item version stating: “Overall, the amount of time I got to spend alone today was”: 1 = *definitely not enough*; 2 = *somewhat less than I would like to*; 3 = *just about right*; 4 = *somewhat more than I would like to*; 5 = *definitely too much*. Higher scores reflected *less* aloneliness.

#### Daily perceived stress

Daily perceived stress was measured through a single face-valid item: “Today, I felt stressed”, reference shifted to experiences across the day but recently used in a study of stress during time spent alone in the pandemic^65^. This is selected instead of the more commonly used perceived stress scale^66^, which operationalizes stress in terms of perceived loss of control over demands, and may be more sensitive to everyday activities that confound with social or solitude time (e.g., business, pressure, deadlines).

#### Day satisfaction

Day satisfaction is a cognitive evaluation of the day derived from life satisfaction, but one that is appropriate for capturing day-level variations^56,67,68^. Participants were asked: “How was your day?”, with a scale ranging from 0 = “*Very Bad*” to 6 = “*Very Good*”. This brief method for assessing satisfaction produces similar results to multi-item measures in past research^69^.

#### Autonomy need satisfaction

Solitude has been described as an opportunity for creativity, self-exploration and connection^18,70–72^ and for these reasons is seen as conducive to autonomy need satisfaction^20^—the experience that one is able to act in a self-congruent way aligned with one’s interests and values^73^. Autonomy need satisfaction was measured using the three-item subscale of the basic psychological need scale^74^, including “I felt free to be who I am”, and “I felt controlled or pressured to be certain ways” (α = 0.70).

### Additional variables

We assessed whether individuals are living alone, with similarly aged friends or family, or cross-generational friends or family (kids or older family members, measured separately). We did not have directional hypotheses about these items.

### Moderators

#### Self-determined motivation

For solitude were measured at the initial session (at the individual level)^35^. The scale (α = 0.81) included seven items that assess seeking solitude for its perceived value. These items included, “It sparks my creativity”, “I enjoy the quiet”, “It helps me get in touch with my feelings”, which were paired with a prompt asking participants: “When I spend time alone, I do so because…”. Therefore, this scale can be understood as assessing one’s general motivation to spend time in solitude because of its perceived value along a number of dimensions.

#### Daily choiceful motivation

At the daily level, we measured choiceful motivation for solitude as a separate predictor of well-being and moderator for effects of time using three randomly selected items per day from an eight-item measure adapted from^17^. The measure asked participants about reasons for which they spend time alone that day. Four items represented choiceful motivation for solitude (e.g., “Because I find the time I spend by myself to be important and beneficial for me”, “Because I simply enjoy the time to be by myself”) and four items represented choiceless motivation (e.g., “Because I was told to be by myself”, “Because I would feel bad about myself if I didn’t do it”). Choiceless motivation items were reverse coded and combined with choiceful motivation items to make up the scores for daily motivation for solitude, with higher scores reflecting greater choiceful motivation for solitude (α = 0.69).

### Analysis plan

The full analysis plan can be found on 10.17605/OSF.IO/5KXQ3. Below we describe the content of those plans that guided the analyses conducted.

#### Missing-at-random analyses

Missing at Random (MAR) were evaluated with a simple regression model predicting each one of our variables from the number of days the study was completed (ranging from 1 to 21 days). We planned to continue to conduct and interpret analyses even if the conditions of MAR were not met; that is, even if the number of days related to any of our variables.

#### Overall primary analytic approach

The primary analyses were conducted in R (Version 1.3.1056). Our Hypotheses 1 and 2 were tested by using hierarchical linear modeling^75^ with nesting within people. We modelled lagged effects that control for previous days’ experiences to provide semi-causal inferences^76,77^. This method is also key to model building because it measures contextual person-level factors (in our case, age and shared within-person variance in our outcomes) simultaneously.

We tested proposed models conducted separately for each of five outcomes (loneliness, perceived stress, aloneliness, autonomy need satisfaction, day satisfaction). We planned to interpret results at *p* < 0.05, but in the "Discussion" section we set out to review which conclusions we had drawn would change were a more conservative^78^ Bonferroni correction be applied with an adjusted benchmark of *p* < 0.01.

#### Unconditional models

Following guidelines^54,79^ for conducting mixed model analyses of diary study data, we first established a series of unconditional models to provide estimates of the fundamental descriptive statistics (mean, within-person variance, and between-person variance) in the proportional time spent alone and well-being outcomes. These unconditional models provided intraclass correlations (ICC^55^, the percentage of total variance in the particular outcome being modeled that is due to between-subject, individual differences at Level 2.

1 $$Day-level \, \left( {within-person} \right):\, y_{ij} = \, \beta_{0j} + \, r_{ij} .$$

2 $$Person-level \, \left( {between-person} \right) :\, \beta_{0j} = \, \gamma_{00} + \, u_{0j} .$$

Here, we nest *i* days within* j* persons with β_0_ representing the mean (for the jth person). The variance of r_ij_ represents the Level 1 (within-person) variance, γ_00_ represents the grand mean (the mean of β across all persons), and the variance of u_0j_ represents the Level 2 (between-person) variance.

#### Quantifying daily balance

Daily balance was operationalized as the proportion of time spent alone relative to socializing.

Linear proportion of time spent in solitude (Ltime):

3 $$Linear \, proportion \, of \, time \left(Ltime\right)= \frac{Hours \, solitude}{Hours \, solitude+Hours \, socializing}$$

Quadratic proportion of time spent in solitude (Qtime):

4 $$Quadratic \, proprotion \, of \, time \left(Qtime\right)=\left(Ltime\right)^2$$

#### Centering

For all models, following guidelines^80^, we group mean centered all measures, including the time-lagged variables. This method of centering ensures that within-person effects are not confounded by between-person differences and is the preferred centering method for examining cross-level interactions and interactions between Level 1 variables^80^.

#### Controlling for lagged effects

To address autocorrelated error structure in the data, we adopted the method utilized by^49^ and create lagged versions of our outcome (well-being) measures. Controlling for these variables accounts for temporal carry-over effects between yesterday’s (i.e., day-1) well-being score on today’s well-being rating.

#### Convergence

We adhered to guidelines^81^ to fit maximal models in all cases where possible. We anticipated that should convergence issues arise, we would drop random effects on a case-by-case basis^82^ by first dropping random correlations close to ± 1 and random slopes of variance close to 0 that are of no essential interest. Although convergence issues are difficult to predict in mixed effect models, preliminary power analyses showed that we had sufficient power to successfully run the proposed random-intercept models. Therefore, our strategy was to start maximally, drop random effects as necessary, and maintain at the very least random-intercept models.

#### Hypothesis 1 tests concerned with time spent in solitude

To test Hypothesis 1 and estimate non-linear slopes in proportional solitude time, we estimated the quadratic slope (when the outcome variable under consideration is either loneliness or perceived stress) or concave down quadratic slope (when the outcome variable under consideration is aloneliness, autonomy need satisfaction, or day satisfaction). The Level 1 (within-person but between-day) tested were modeled as:

5 $$Day \, Level \, \left( {Within \, Person} \right): y_{ij} = \, \beta_{0j} + \, \beta_{1j} \left( y \right)_{i - 1j} + \, \beta_{2j} \left( {Ltime} \right)_{ij} + \, \beta_{3j} \left( {Qtime} \right)_{ij} + \, r_{ij.}$$

At Level 2 (between-person) the linear and quadratic portions of time spent in solitude across the 21-day duration were simultaneously modeled as:

6 $$\begin{aligned} Person - Level{\mkern 1mu} \left( {between - person} \right):\,  &\beta _{{0j}} = {\mkern 1mu} \gamma _{{00}} + {\mkern 1mu} \gamma _{{01}} \left( {Ltime} \right)_{j} + {\mkern 1mu} \gamma _{{02}} \left( {Qtime} \right)_{j} + {\mkern 1mu} u_{{0j}} \\ & \beta _{{{\text{1j}}}} = \gamma _{{10}} + u_{{1j}} \\ & \beta _{{2j}} = {\mkern 1mu} \gamma _{{20}} + {\mkern 1mu} u_{{2j}} \\& \beta _{{3j}} = {\mkern 1mu} \gamma _{{30}} + {\mkern 1mu} u_{{3j}} \\ \end{aligned}$$

These models nested *i* days within *j* persons, with r_ij_ representing the Level 1 (within-person) variance, γ_00_ representing the grand mean, and u_0j_ representing the Level 2 (between-person) variance. At Level 1, models controlled for the lagged outcome variable of the previous day (*day-1* of *y*) to account for potential temporal carry-over effects. As such, the slope coefficient *γ*_*10*_ represents the mean (across the sample) association between yesterday’s well-being value and today’s (concurrent) wellbeing rating, with *u*_*1j*_ representing the extent to which person _*j*_ differs from the sample mean (random slope). *γ*_*20*_ and *γ*_*30*_ represent the mean association between the proportion of time spent in solitude (linear and quadratic proportions respectively) and the well-being rating, with *u*_*2j*_ and *u*_*3j*_ representing the random slopes.

Providing we did not find a statistically significant quadratic slope at Stage 1, we planned to interpret the linear slope of time, which is derived from Stage 1 analysis alongside the quadratic slope. We planned to take this alternate path (focusing on linear vs. quadratic effects) at both Levels 1 (daily) and 2 (individual) effects. We anticipated that if we find neither a significant quadratic nor linear effect, we could conclude there is no evidence of a general solitude time effect on well-being at either or both the daily (Level 1) or individual (Level 2) levels.

#### Hypotheses 2–4 tests concerned with motivation effects

The following model (Eq. 7) tested Hypothesis 2 concerning the main effects of *choiceful* motivation and take the first step to test Hypothesis 3 concerning its moderating effects on time. The implicit nesting of days within people remains and our model took into consideration random effects of these day-level predictors.

7 $$Day-Level \, \left( {Within \, Person} \right): y_{ij} = \, \beta_{0j} + \, \beta_{1j} \left( y \right)_{i - 1j} + \, \beta_{2j} \left( {Ltime} \right)_{ij} + \, \beta_{3j} (Qtime_{ij} + \, \beta_{4j} \left( {Choice} \right)_{ij} + \, \beta_{5j} \left( {Choice_{ij} * \, Ltime_{ij} } \right) \, + \, \beta_{6j} \left( {Choice_{ij} * \, Qtime_{ij} } \right) \, + \, r_{ij. }$$

Further, to test the main effects of *self-determined motivation* at an individual level, and take a first step to testing its moderating effect on time in solitude, we tested models (8) and (9), now focusing on Level 2 main and moderation effects:

8 $$Day-Level \, \left( {Within \, Person} \right): y_{ij} = \, \beta_{0j} + \, \beta_{1j} \left( y \right)_{i - 1j} + \, \beta_{2j} \left( {Ltime} \right)_{ij} + \, \beta_{3j} \left( {Qtime} \right)_{ij} + \, r_{ij }$$

9 $$\begin{aligned} Person - Level{\mkern 1mu} \left( {between - person} \right):\,  \beta _{{0j}} = {\mkern 1mu} \gamma _{{00 + }} \gamma _{{01}} \left( {{\text{sdMot}}} \right)_{j} + u_{{0j}} \\  \beta _{{{\text{1j}}}} = \gamma _{{10 + }} u_{{1j}} \\  \beta _{{2j}} = {\mkern 1mu} \gamma _{{20 + }} \gamma _{{21}} \left( {{\text{sdMot}}} \right)_{j} + u_{{2j}} \\  \beta _{{3j}} = {\mkern 1mu} \gamma _{{30 + }} \gamma _{{31}} \left( {{\text{sdMot}}} \right)_{j} + u_{{3j}} \\ \end{aligned}$$

In models 8 and 9, γ_00_ represents the fixed intercept (grand mean across all persons and days), γ_01_ represents the main effect of self-determined motivation for solitude for a given individual on wellbeing, and u_0j_ represents the person-level residual. As before, *γ*_*10*_ represents the mean (across the sample) association between yesterday’s well-being value and today’s (concurrent) well-being rating. γ_20_ and γ_30_ represent fixed slopes (mean slopes across all persons and days) for the proportion of linear time and quadratic time spent in solitude respectively. The slope coefficients γ_21 and_ γ_31_ represent whether the association between time spent in solitude (linear or quadratic proportions respectively) was moderated by an individual’s self-determined motivation for solitude. Random slopes are represented by u_1j_ − u_3j_.

We planned that, providing we did not find a statistically significant quadratic or linear slope when testing Hypothesis 1 at Stage 1, we would still test for moderation effect by choice (Level 1) or self-determination (Level 2). This is because even if there are no main quadratic or linear effect across the sample, different levels or patterns of effects  might still be in evidence for those high or low in motivation. As an example, a motivation X linear slope interaction could show that those high in autonomous motivation benefit from solitude time in terms of greater well-being, whereas for those low in autonomous motivation, the more time in solitude the lower well-being they report (a negative effect). Similarly, although we hypothesized directional relational of simple main effects of solitude time above and below the minima/maxima on well-being when testing H1, we planned to continue to test statistically significant interaction effects even if we did not find support for these directional hypotheses. We describe significant interactions below in line with any statistically significant patterns observed when conducting these tests and discuss how they met or did not meet the assumptions made.

## Results

### Planned missing-at-random analyses

We evaluated whether data were Missing at Random (MAR) with a simple regression model predicting each one of our variables from the number of days the study was completed (ranging from 1 to 21 days). Analyses did not show relations between linear time in solitude, quadratic time in solitude, or self-determined motivation for solitude in relation to days completed (βs < 0.06, *ps* > 0.860). Further, days completed did not relate to any of the five well-being indicators (βs < 0.08, *ps* > 0.494), or choiceful motivation for solitude (β = 0.05, *p* = 0.618).

### Exploratory trends across time

We first examined whether there were any trends over time in the outcome variables. To do so, we estimated a multilevel regression model of each outcome on the day of study with random intercepts and slopes over participants. Both day satisfaction and autonomy need satisfaction slightly decreased over time (satisfaction *b*_day_: −0.02, 95% CI [−0.03, −0.01], *p* < 0.001; autonomy *b*_day_: −0.01, 95% CI [−0.02, 0.00], *p* = 0.014), but neither loneliness (*b*_day_: 0.00, 95% CI [−0.01, 0.01], *p* = 0.934), aloneliness (*b*_day_: 0.00, 95% CI [−0.01, 0.00], *p* = 0.483), or stress (*b*_day_: −0.01, 95% CI [−0.02, 0.00], *p* = 0.170) showed significant trends.

### Planned intraclass correlations

Intraclass correlations were similar across outcomes (day satisfaction: 0.37, 95% CI [0.29, 0.43]; loneliness: 0.44, 95% CI [0.36, 0.51]; aloneliness: 0.31, 95% CI [0.25, 0.37]; stress: 0.34, [0.24, 0.41]; autonomy need satisfaction: 0.46, 95% CI [0.40, 0.52]). Together, they indicated that approximately 31% to 46% of the variance occurred between, rather than within, individuals.

### Exploratory scatterplots

**Figure 1:**
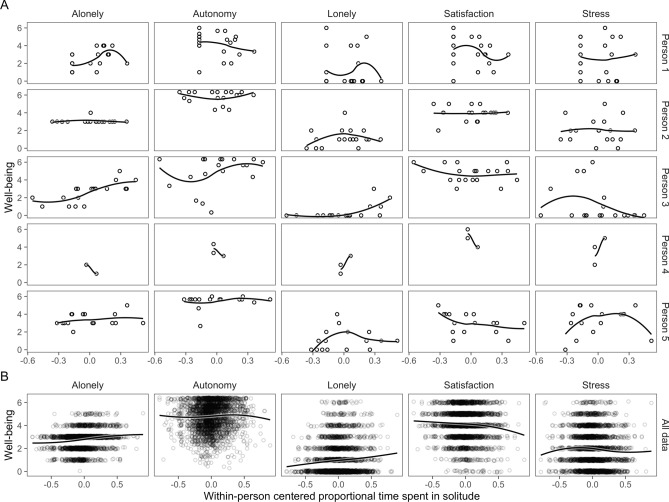
Scatterplots of five well-being outcomes on proportional time spent in solitude. (**A**) Scatterplots for three random participants. (**B**) Scatterplots of all data. Lines are nonparametric exploratory LOESS fits.

Before presenting our focal hypothesis tests, we present unplanned scatterplots of the raw well-being outcomes on proportional time spent in solitude in F1. In addition to the data, F1 shows locally weighted smooths (LOESS) that describe central tendencies in the well-being outcomes as functions of proportional time spent in solitude, without imposing parametric constraints on the association. Visual inspection did not immediately suggest any strong trends in those associations (Fig. 1).

### Primary registered tests

Results are presented separately, following interpretations as outlined in Table 1.

**Table 1:** Key parameter estimates.

Parameter	Day satisfaction	Day loneliness	Day aloneliness (r)	Daily stress	Daily autonomy
Within-person effects					
Linear time in solitude	**−0.47 (−0.71, −0.23),* p* < 0.001**	**0.61 (0.36, 0.86),* p* < 0.001**	**0.71 (0.55, 0.87), *p* < 0.001**	**−0.42 (−0.68, −0.16), *p* = 0.002**	**0.32 (0.12, 0.53), *p* = 0.002**
Quadratic time in solitude	−0.03 (−0.78, 0.71),* p* = 0.928	0.18 (−0.57, 0.93),* p* = 0.629	0.09 (−0.33, 0.52), *p* = 0.665	−0.58 (−1.51, 0.35), *p* = 0.219	−0.01 (−0.72, 0.70), *p* = 0.971
Daily choiceful motivation	**0.51 (0.41, 0.62),* p* < 0.001**	**−0.41 (−0.52, −0.30),* p* < 0.001**	−0.05 (−0.12, 0.02), *p* = 0.145	**−0.51 (−0.63, −0.38), *p* < 0.001**	**0.39 (0.29, 0.50), *p* < 0.001**
Linear time X daily choiceful motivation	**0.49 (0.17, 0.80),* p* = 0.003**	**−0.34 (−0.64, −0.03),* p* = 0.032**	−0.06 (−0.28, 0.17), *p* = 0.625	0.03 (−0.33, 0.40), *p* = 0.853	0.20 (−0.07, 0.48), *p* = 0.152
Quadratic time X daily choiceful motivation	−0.22 (−1.05, 0.60),* p* = 0.592	0.11 (−0.73, 0.96),* p* = 0.790	−0.33 (−0.93, 0.27), *p* = 0.280	−0.27 (−1.29, 0.76), *p* = 0.611	0.16 (−0.56, 0.87), *p* = 0.668
Within-person X between-person effects					
Linear time (Level 1) X self-determined motivation (Level 2) interaction	0.20 (−0.02, 0.42), *p* = 0.073	−0.12 (−0.34, 0.10), *p* = 0.298	−0.05 (−0.19, 0.09), *p* = 0.495	−0.17 (−0.40, 0.07), *p* = 0.171	0.07 (−0.11, 0.25), *p* = 0.426
Quadratic time (Level 1) X self-determined motivation (Level 2) interaction	−0.04 (−0.70, 0.63), *p* = 0.915	−0.37 (−1.03, 0.28), *p* = 0.264	−0.25 (−0.63, 0.13), *p* = 0.197	0.13 (−0.71, 0.98), *p* = 0.754	−0.22 (−0.88, 0.43), *p* = 0.504
Between-person effects					
Linear time	0.10 (−0.50, 0.71), *p* = 0.737	−0.02 (−0.70, 0.66), *p* = 0.954	**0.78 (0.48, 1.08), *p* < 0.001**	**−1.11 (−1.81, −0.41), *p* = 0.002**	**0.80 (0.20, 1.40), *p* = 0.009**
Quadratic time	−1.07 (−2.98, 0.84), *p* = 0.273	1.63 (−0.54, 3.81), *p* = 0.140	−0.13 (−1.08, 0.82), *p* = 0.790	1.68 (−0.56, 3.92), *p* = 0.141	−1.41 (−3.30, 0.49), *p* = 0.146
Self-determined motivation	0.05 (−0.07, 0.17), *p* = 0.379	**0.16 (0.02, 0.29),** ***p = 0.023***	−0.03 (−0.10, 0.03), *p* = 0.297	0.12 (−0.02, 0.26), *p* = 0.097	−0.04 (−0.16, 0.08), *p* = 0.526

“Linear time” and “quadratic time” refer to the linear and quadratic associations between proportional time (in terms of hours per day) spent in solitude and the outcome defined at the top of the table. “Choiceful motivation” is measured as a scale at the day-level, and “self-determined motivation” is its parallel construct measured at the individual level. Aloneliness is reversed, such that higher scores reflect less aloneliness. Parameters with “X” indicate an interaction. The numbers indicate point estimates, 95% CIs in parentheses, and two-sided *p*-values.

Significant values are in bold.

#### Hypothesis 1

**Figure 2:**
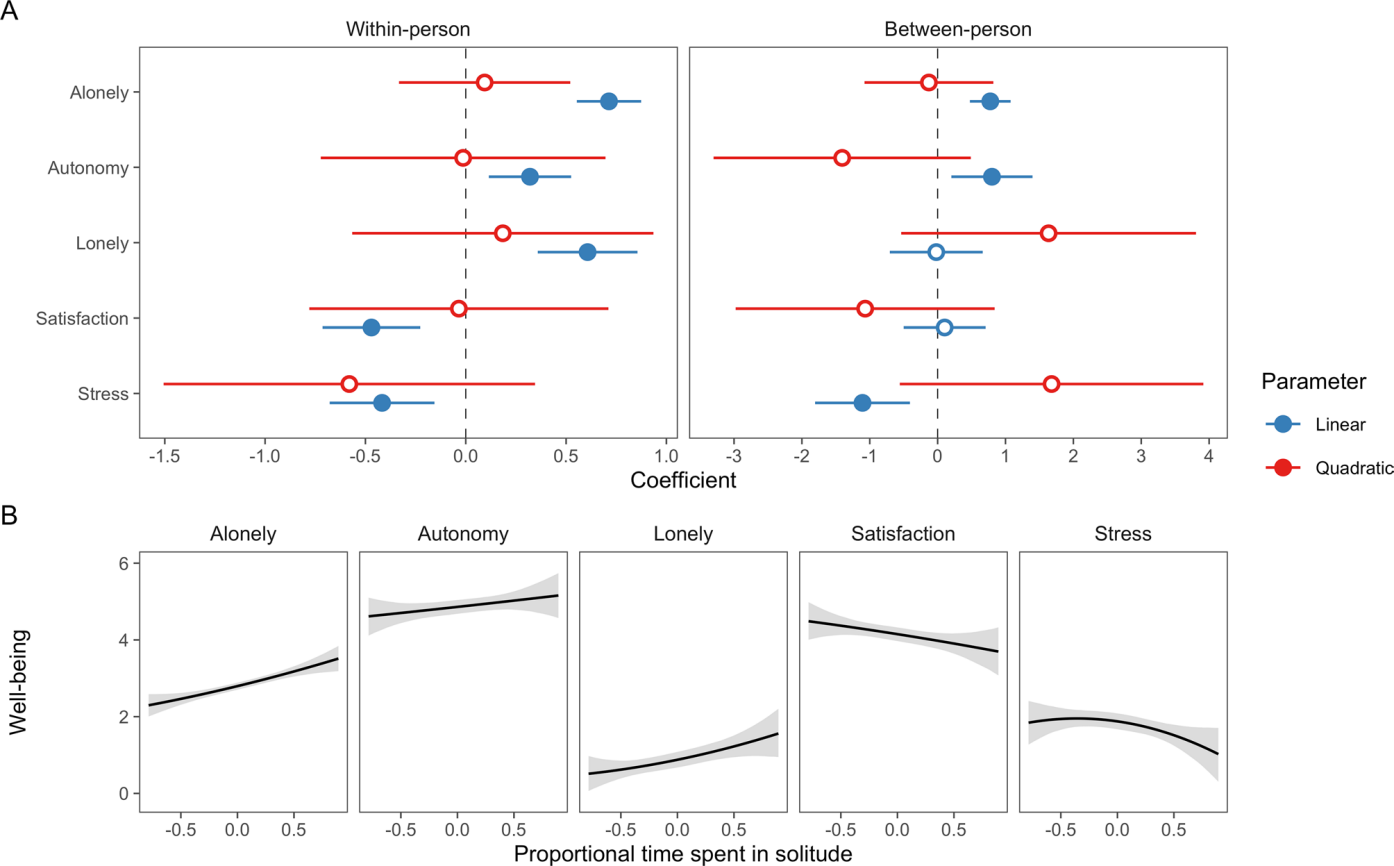
Parameter estimates (**A**) and fitted lines (**B**) from Model 1 targeting research questions 1 and 2. Intervals are 95% confidence intervals. Filled points indicate coefficients significant at the 0.05 level.

Nested models tested Research Question 1 concerning the (potentially quadratic) associations between time spent in solitude and well-being. Figure 2A show parameter estimates from multilevel models fitted separately to each outcome in accord with the analysis plan.

We hypothesized (H1) quadratic associations between time spent in solitude and well-being, but this hypothesis was not supported: none of the quadratic associations were statistically significant (Fig. 2A, quadratic parameters; see also Table 1). Our plan was that should we not find a statistically significant quadratic slope at Stage 1, we would interpret the linear slope of time, which is derived from Stage 1 analysis alongside the quadratic slope. Doing this, we found that time spent in solitude related to greater autonomy need satisfaction and loneliness, and to less aloneliness, day satisfaction and stress. For example, for the average person, days in which all time was spent in solitude were associated with approximately 0.5 scale units of lower day satisfaction than those days in which no time was spent in solitude. At the between-person level, we found that average time spent in solitude averaged across the duration of the study (between 3 and 21 days) predicted average autonomy need satisfaction positively, and less aloneliness and stress, but again we found no significant quadratic associations (RQ2). The resulting model-estimated associations are illustrated in Fig. 2B and summarized in Table 1.

### H2–H4: motivation for solitude

**Figure 3:**
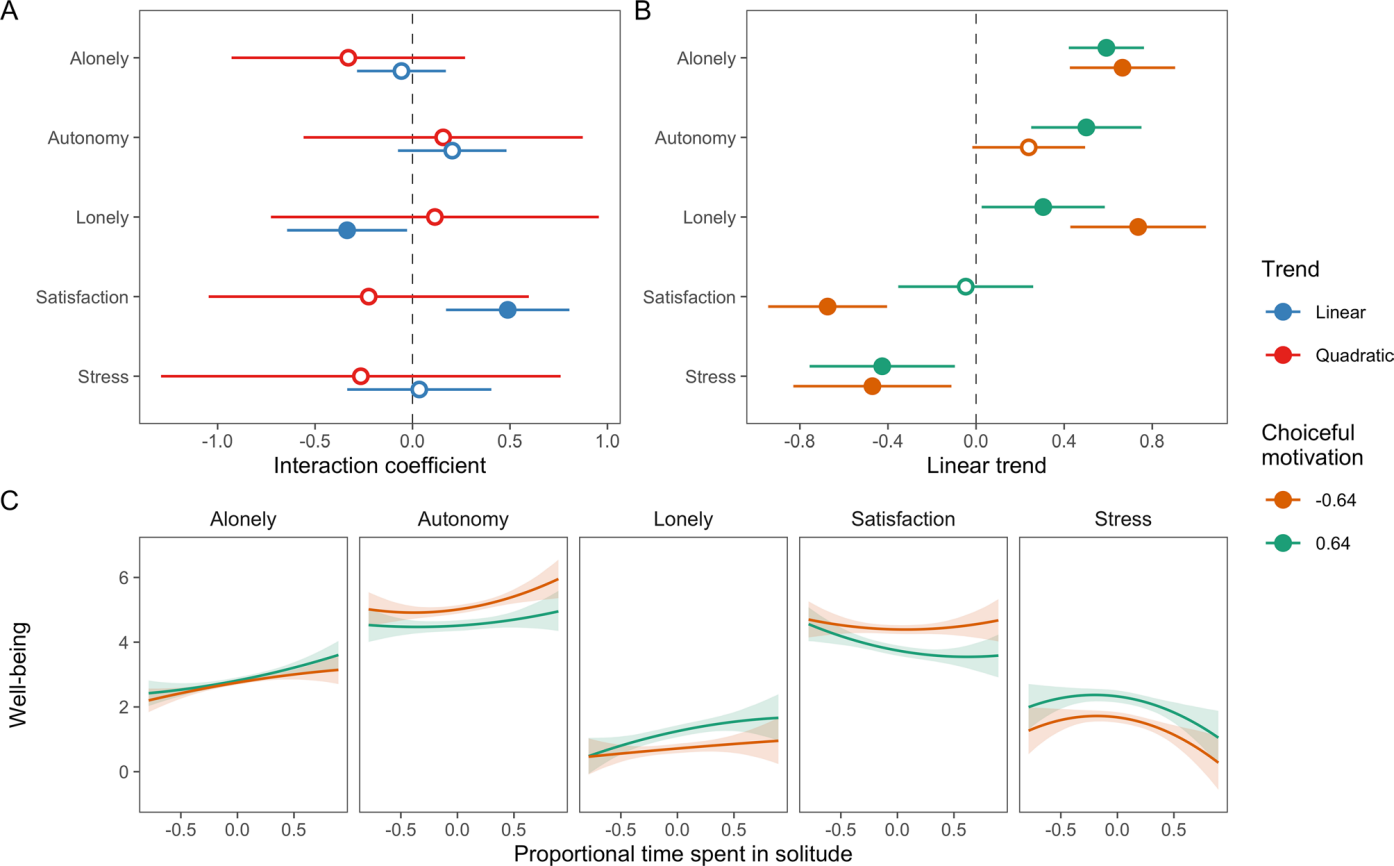
The moderating effects of daily choiceful motivation for solitude on associations between proportional time spent in solitude and well-being. (**A**) Estimated interaction coefficients between linear and quadratic solitude time trends and choiceful motivation with 95% CIs. Filled points indicate significant coefficients. (**B**) Linear associations between solitude time and well-being for different levels of choiceful motivation. (**C**) Model-estimated associations between time spent in solitude and well-being for − 1SD and + 1SD of choiceful motivation.

Findings testing H2 to H4 showed that daily choiceful motivation for solitude related to greater day satisfaction and autonomy need satisfaction, and to lower loneliness and stress (Table 1). We did not find different quadratic trends between different levels of daily choiceful motivation (Fig. 2). However, the linear trends between solitude time with loneliness and day satisfaction differed between levels of daily choiceful motivation. On days that were relatively high on choiceful motivation, the association between solitude time and day satisfaction was small and non-significant; on days low on choiceful motivation, increased solitude time was significantly associated with lower day satisfaction. Similarly, when choiceful motivation was low, increased time in solitude was positively related to loneliness; this effect was less pronounced when choiceful motivation was high (Fig. 3).

**Figure 4:**
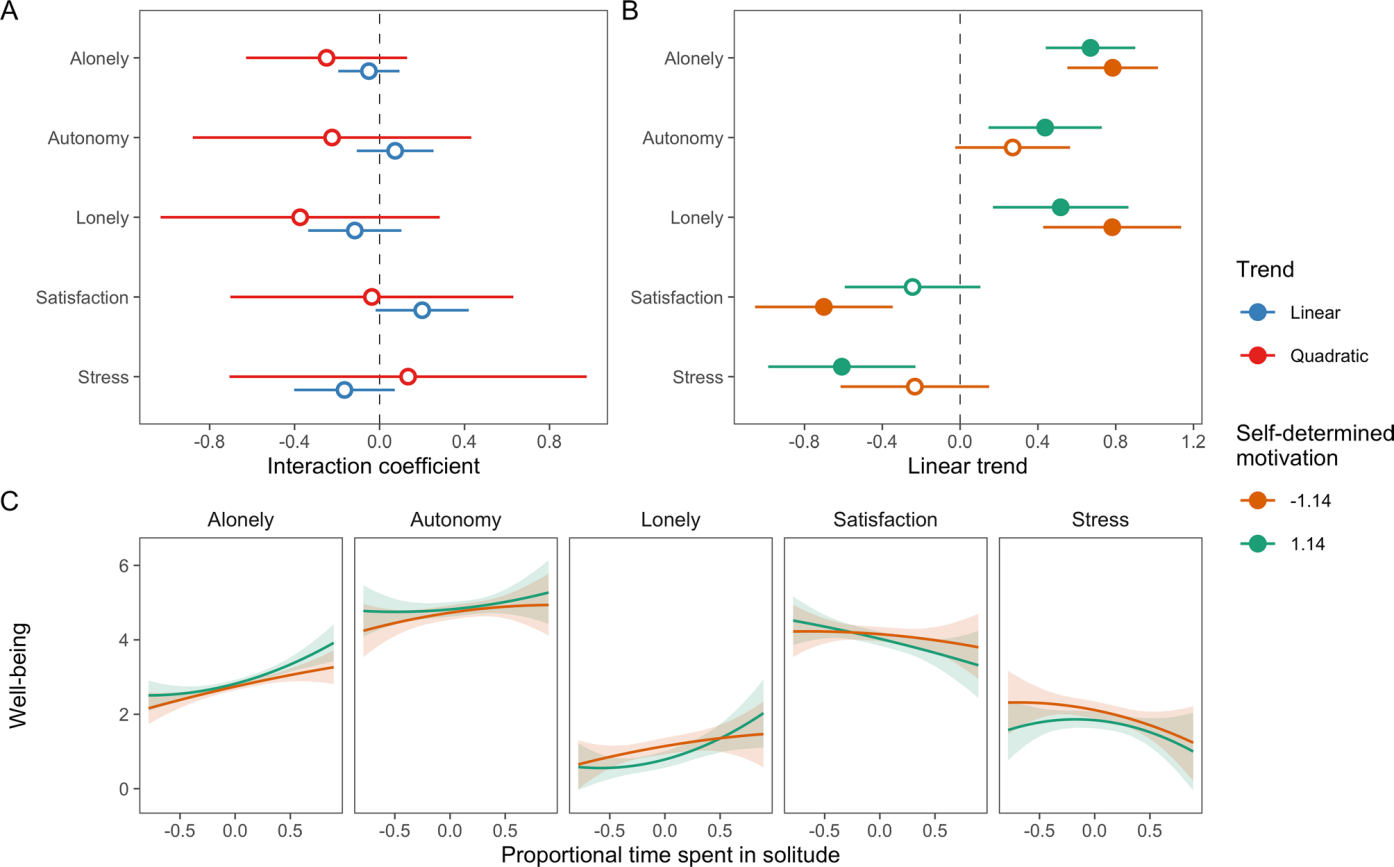
The moderating effects of person-level self-determined motivation for solitude on associations between proportional time spent in solitude and well-being. (**A**) Estimated interaction coefficients between linear and quadratic solitude time trends and self-determined motivation with 95% CIs. Filled points indicate significant coefficients. (**B**) Linear associations between solitude time and well-being for different levels of self-determined motivation. C. Model-estimated associations between time spent in solitude and well-being for −1SD and + 1SD of self-determined motivation.

We then examined the moderating effects of self-determined motivation. First, we found that self-determined motivation related to loneliness positively across individuals, but no other associations were significantly different from zero (Table 1). Moreover, there were no significant interaction terms between self-determined motivation and either linear or quadratic associations between time spent in solitude and well-being (Fig. 4).

## Discussion

This study was built on the view that while solitude’s effects on well-being have been viewed in broadly negative and broadly positive terms, the role that it plays in daily life may depend on how it is balanced alongside social activities: too little solitude deprives individuals of time to relax and reconnect to their selves^7^, whereas too much time alone can be isolating and lonely^24^. We expected that, perhaps, the *right* amount of solitude in daily life would provide the opportunities solitude presents, while also protecting from potential well-being costs. To determine that right amount of solitude, we used a day reconstruction method and calculated the proportional time spent in solitude using the numbers of hours in solitude versus non solitude episodes for each day. This allows us to estimate the amount of time spent in solitude relative to time spent in social interactions.

Confirmatory findings did not find evidence to support a ‘balance’ model of solitude based on daily hours of time alone or in social activities. Specifically, our study did not find evidence of quadratic functions that suggest tipping points at either the between-person and within-person level. That means these findings did not support a model wherein a ‘one-size-fits-all’ number of hours when solitude time stopped being beneficial and social time started benefiting (or vice versa).

Instead, the proportional time spent in solitude was best understood in linear terms, and these effects were nuanced. A first finding indicated that as individuals spent more time in solitude on a given day, they were less satisfied by, and lonelier during, that day. These linear trends are in line with other daily diary research showing that solitude time can be lonelier and less satisfying^40^. This study provided additional support that time spent in daily solitude can reduce some aspects of daily well-being. But costs of solitude observed in this study were not straightforward: instead, they were moderated by individuals’ choiceful motivation for solitude on that day. Specifically, on days in which participants spent larger quantities of time alone but felt more choiceful in their solitude, there was no cost to their day satisfaction; they felt just as satisfied as if they had spent less time in solitude. And the cost in terms of their increased loneliness was lessened.

Given the number of outcomes tested meant the use of multiple models, we planned to interpret findings for a second time using a Bonferroni correction as a lens for a more conservative approach. From the current findings, only the interaction between time spent in solitude and choiceful motivation on loneliness would become non-significant, in other words, it may be that time spent in solitude relates to greater loneliness regardless of the level of choiceful motivation one feels for this time.

### Implications

These findings are consistent with a previous study^41^ showing that autonomous motivation reduces the well-being cost of solitude time at a between-person level, but here we observed that it is not a general tendency to be autonomous (i.e., choiceful) in solitude but rather benefits were driven when individuals felt choiceful for those days’ solitude, suggesting a role for daily contextual drivers that increase or decrease individuals choosing solitude to a greater extent on any particular day. Similarly, alongside our empirical models, intraclass correlations computed for both loneliness and aloneliness helped to understand the role of a day’s experiences in solitude-seeking. Approximately 44% of the variance in loneliness and 31% of the variance in aloneliness existed at individual levels, suggesting that at least half or even a majority of the shifts in both experiences varied substantially *within* individuals from day to day. These findings indicate that both feeling lonely (wanting more connection than one has) and also feeling alonely (wanting more solitude than one has) is responsive to occurrences on that day and the corresponding daily needs of individuals to be alone or alternatively to connect with others.

It would be fascinating in future research to explore what those daily drivers may be. For example, it may be people seek solitude choicefully when feeling especially creative or self-reflective^9^. The findings also support an increasing body of research that has suggested direct links between choiceful motivation for solitude and well-being^17,20,35^, but current findings build on it by evidencing that choiceful motivation can shape the way that *extended* time in solitude impacts well-being. In other words, it reduces the sense of ‘too much’ solitude that transforms solitude into social isolation^83^.

Also noteworthy was that these linear relations of solitude time with both loneliness and lower day satisfaction were not evident when time in solitude was averaged across the duration of the study. These models suggested that that more time spent in solitude correlates with reduced day satisfaction and increased loneliness only at the within-person level but not at the between-person level. Whereas spending more time in solitude than normal on a particular day was associated with less satisfaction and more loneliness, those who generally spent more time alone did not necessarily experience lower global well-being. This finding lies in contrast with a common stereotype that people who are alone more frequently are ‘lonely people’^84^. Instead of supporting a ‘lonely person’ model, well-being detriments of extended solitude were only in evidence when a person spent an unusual and greater amount of their time in solitude than was normal for them.

Also unexpected, global self-determined motivation for solitude did not moderate effects of time on either level of analysis (day or person), suggesting that occurrences on a given day may influence loneliness and day satisfaction more so than there being a particular ‘capacity’ for solitude determined by a penchant or valuing for time alone^70^. Importantly, in this study we have only explored one individual difference (self-determined motivation) that may impact responses to solitude. Other individual differences such as attachment style^85^, dispositional autonomy^86^, or even more immediate—a capacity for solitude construct^87^—were not tested in this study and may play more central roles in daily well-being.

Although solitude time came with particular well-being costs, it also correlated with other well-being *benefits*. Specifically, on days in which people spent more time in solitude, they reported feeling less stress during that day. Paralleling this on an individual-difference level, those who spent more time alone across the duration of the study were generally lower in stress across the days of the study. These findings speak to solitude time’s deactivation effect, the ability of solitude to reduce high-arousal emotions (such as anxiety) and breed low arousal emotions (including feelings of calm and peacefulness)^17,40,88–90^, but they counter other daily data that suggests solitude time is associated with greater cortisol levels, that are often, but not solely indicative of stress^91^. The questions of how solitude helps promote a sense of peace and relaxation, or perhaps whether it can help to regulate daily life stress and stressors^92^, are important ones for further study to understand the functional benefits of affective deactivation in daily life. For example, researchers can explore whether calming qualities of solitude help individuals to cope with daily stressors or alternatively it might be that daily stressors interfere with the solitude time–lower stress link to the extent that people cope with stress better in social environments^93^.

In our study, reduced stress was not the only beneficial correlate of time spent alone. On days in which individuals spent more time in solitude, and those individuals who spent more time in solitude across the duration of the study, reported greater daily autonomy need satisfaction, the experience of being volitional and choiceful, and free of controls and pressures to be certain ways^94^. This finding supports conceptualizations that solitude offers an opportunity for self-exploration and self-connection^18,70–72^, presumably because it gives individuals the space to pursue activities in which they are truly interested in or want to do. When in solitude, there is less of a need to compromise or negotiate how time is spent, and individuals can be freed from social pressures and expectations^20^. The current findings support these views, suggesting that daily solitude time may confer particular self-connection and personal volition opportunities.

Considering the moderating role of autonomous motivation for solitude, although the detrimental relations between solitude time and increased loneliness and reduced day satisfaction were mitigated when solitude was choicefully (i.e., autonomously) motivated, the *beneficial relations* between time spent in solitude and reduced stress as well as higher autonomy need satisfaction were not further affected by either daily or individual-level autonomous motivations. In other words, quite surprisingly choiceful motivation for solitude did not seem to improve, or detract from, either a lack of stress or a sense of being autonomy need satisfaction that was associated with time spent in solitude.

### Limitations and future directions

The current study set out to test whether solitude and social interactions each makes proportional contributions to daily well-being. We did not find evidence to support the ‘goldilock effect’ that too much or too little *quantity* of solitude relative to time spent in social interactions would impact well-being differently when compared to a definable balance between the two. It is possible that balance might not be established through defining the quantity of solitude and social interactions, but rather through considering the quality of each of these states.

There are a number of intriguing possibilities for defining the quality of social and solitude experiences, in balance. First, the reasons we choose or are forced to be in either context drives how each influences us^95^, and while we measured reasons for solitude we did not measure reasons for social time. In addition, *what we do* when we are alone, and who we are with when we are in social contexts (for example, family vs. romantic partner vs. friend vs. work colleague) influences daily well-being^96,97^. Finally, engaging in different solitary activities (e.g., leisure vs. work vs. meditating vs. ruminating; with many different possibilities for taxonomies) may have implications for well-being within solitude and solitude’s contribution to well-being across a day^98^. By zooming in on specific qualities of time alone and in social contexts, future work can meaningfully develop an understanding of balance.

Further, our current study defined solitude in terms of time spent alone and not interacting with others through virtual technology (e.g., text messaging, social media), and the question remains whether virtual interactions impact balance and well-being in positive or negative ways. Some evidence to date suggests that computer-mediated interaction takes place during a substantial amount of the time spent alone, particularly for young people^98^. Although virtual interaction does not seem to affect whether or not a person would consider themselves to be alone^60^, the inclusion of social media in our operationalization of solitude in this sample might compromise our ability to truly distinguish between different types of social time (in-person or virtually).

Further, the sample itself can be more targeted to understanding balance as it occurs for individuals in a specific age range. Our current study examined balance in adult aged over 35 years, but did not differentiate developmental periods across early, middle, or late adulthood. These stages are meaningful; during these periods well-being improves in important ways^99^, as does the relationship with solitude itself, which is felt to be more peaceful and rewarding in older adults than in early adulthood^39,100^. Yet some relevant observations generalize across age groups; for example, broadly speaking individuals experience more positive affect in solitude when they have close relationships within their everyday lives^40^. The present results cannot be generalized to any particular age group with confidence and further study is needed to understand how time alone and with others—in relation to one another—affect individuals within specific developmental periods.

Although the day construction method offers a structured way to quantify solitude hours during a day, reducing retrospective bias^58^, this method is not capable of eliminating it altogether, and the analyses presented in this study were estimated at the day-level using end of day reports of both the outcomes under study and choiceful motivation for solitude. Tighter controls over how participants spend spans of time alone or with others ranging from 15 min to a few days can help to more sensitively test the pathways we conceptualized, for example linking time alone to loneliness.

In a similar vein, the study design produced tests that were in effect correlational, and while we anticipated that time in solitude would confer certain benefits (or hold certain costs) to daily well-being, there is no evidence to support these causal interpretations. For example, it is plausible that the link between time spent alone and day satisfaction as well as loneliness has to do with a lack of alternative activity options or social connections that pull time away from solitude. Future research developing these pathways through time-lagged models and experimental interventions would be fruitful for addressing these issues.

In conclusion, this study suggested that ‘balance’ is not best conceptualized in terms of hours of solitude time. Quantified time in solitude relative to social interactions may fail to capture balance, and we did not find evidence that there is a “golden” standard for how much time spent in solitude to benefit from it. Despite these findings, the importance of having balance between social and solitude time has been expressed in participants’ accounts from qualitative data^60^. Other measures of balance may be more suitable, and indeed solitude may better be understood as a subjective, not an objective, state. The present study found that on days when more time is spent in solitude, the abundance of it may confer both positive and negative benefits for our daily experiences. Understanding those costs and benefits of solitude, and also which qualities of solitude may benefit well-being, can inform perceptions of ‘balance’ and build knowledge that helps us to capitalize on time spent alone.

## Data Availability

Deidentified data for the study can be accessed at https://osf.io/zx7ms/.

## Code availability

Study code can be accessed at https://osf.io/zx7ms/ .
